# Infection with *Angiostrongylus cantonensis* reveals up- and down-regulation of the protein profile in the mucus of infected slugs

**DOI:** 10.1017/S0031182025000435

**Published:** 2025-04

**Authors:** Joana Borges Osório, Giovanni Gosch Berton, Leandro Mattos Pereira, Jeremy Potriquet, Renata Russo F. Cândido, Jason Mulvenna, Malcolm K. Jones, Carlos Graeff-Teixeira, Alessandra Loureiro Morassutti

**Affiliations:** 1 School of Health Sciences, Pontifical Catholic University of Rio Grande do Sul, Porto Alegre, Brazil; 2 School of Medicine, University of Passo Fundo, Passo Fundo, Brazil; 3 School of Medicine, University of Padova, Padua, Italy; 4 Interdisciplinary Centre of Marine and Environmental Research (CIIMAR), University of Porto, Matosinhos, Portugal; 5 Queensland Institute of Medical Research, Berghofer Medical Research Institute, Brisbane, QL, Australia; 6 School of Veterinary Science, The University of Queensland, Gatton, QL, Australia; 7 Department of Pathology, Federal University of Espírito Santo, Vitória, Brazil; 8 Postgraduate Program in Dentistry, University of Passo Fundo, Passo Fundo, Brazil

**Keywords:** biomarkers, host–parasite relationship, mollusc infection, non-invasive diagnosis, proteomics

## Abstract

Host–parasite adaptation is crucial for evolutionary success of a parasite. The nematode *Angiostrongylus cantonensis* has a heterogenic life cycle involving molluscs as intermediate hosts and rats as definitive hosts. Several mollusc species are susceptible hosts of *A. cantonensis*, allowing the development of first-stage larvae (L1) into third-stage larvae (L3). Changes in the metabolism of infected molluscs have been demonstrated, disturbing regular routes and inducing host defence mechanisms. This study aimed to identify changes in the proteomic content of *Phyllocaulis* spp. mucus during *A. cantonensis* infection. Proteins were extracted from the mucus samples of infected and non-infected slugs and identified using liquid chromatography–tandem mass spectrometry. We found 26 up-regulated and 15 down-regulated proteins in infected slugs compared to non-infected slugs. Protein profiles are promising markers of parasite infection, and a better understanding of proteomic profiles during infection may help inform *in vivo* studies and promote new tools for the non-invasive identification of infected hosts.

## Graphical abstract

**Figure:**
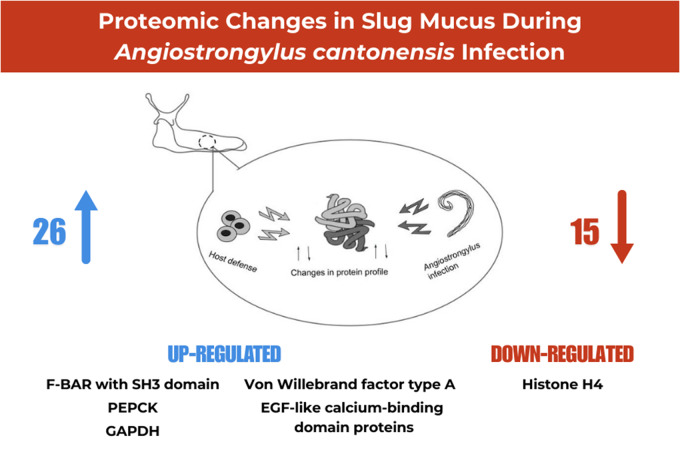

## Introduction

Understanding the interactions between parasites and their hosts is fundamental to elucidating the mechanisms of disease transmission and developing control strategies. The nematode *Angiostrongylus cantonensis*, the causative agent of eosinophilic meningitis in humans, has a complex life cycle involving molluscs as intermediate hosts and rats as definitive hosts (Wang et al., 2012). Various slug and snail species can serve as intermediate hosts, supporting the development of first-stage larvae into infective third-stage larvae (Caldeira et al., 2007; Chan et al., 2015).

Molluscs from the Veronicellidae family, such as *Phyllocaulis* spp., are known to be susceptible to *A. cantonensis* and are utilized for life cycle maintenance in laboratory settings (Graeff-Teixeira et al., 1993; Morassutti et al., 2014). Molluscs secrete complex mucus rich in glycoproteins, enzymes and antimicrobial peptides, which play roles in locomotion, defence and environmental adaptation (Qvarnstrom et al., 2007). The biological potential of molluscan mucus has been explored for therapeutic and cosmetic applications because of its antimicrobial and anti-inflammatory properties (Tsvetanova et al., 2020; Rizzi et al., 2021).

Infection with *A. cantonensis* can modulate the immune response and metabolic pathways of molluscs, leading to alterations in protein expression and oxidative stress responses (Mendes et al., 2020). Investigating these proteomic changes could reveal biomarkers for infection and enhance our understanding of host–parasite dynamics.

This study aimed to analyse the proteomic alterations in the mucus of *Phyllocaulis* spp. experimentally infected with *A. cantonensis*, to identify potential biomarkers for infection and to contribute to the development of non-invasive diagnostic tools.

## Materials and methods

### Collection of molluscs and maintenance of the parasite lifecycle

*Phyllocaulis* spp. slugs were collected in Porto Alegre, Brazil, and reared in laboratory conditions at Pontifícia Universidade Católica do Rio Grande do Sul (PUCRS). The slugs were maintained in plastic containers with garden soil and fed fresh vegetables *ad libitum*.

### Phyllocaulis *spp. infection and collection of mucus*

Ten slugs were divided into 2 groups: a control group (CG) and an infected group (IG). IG was exposed to approximately 6,000 first-stage larvae of *A. cantonensis* from the Vila Fátima isolate (Cognato et al., 2013). After a 30-day infection period, mucus samples were collected by gently scraping the slug bodies with a moistened swab. The samples were stored at −80 °C until analysis. Infection was confirmed by artificial digestion of slug tissues and microscopic examination of the larvae.

### Proteomic experiment

Protein extraction from mucus samples was performed using a modified filter-aided sample preparation method (Potriquet et al., 2017). Proteins were reduced, alkylated and digested with trypsin. The peptides were purified and concentrated using ZipTip C18 pipette tips.

Liquid chromatography–tandem mass spectrometry was conducted using a Shimadzu Prominence Nano High-Performance Liquid Chromatography coupled with a TripleTOF 5600+ mass spectrometer (AB SCIEX). Data acquisition was performed using Information Dependent Acquisition, and data analysis was performed using Analyst 2.0 software.

Protein identification was accomplished by searching spectral data with 2 independent software programmes: X! Tandem and ProteinPilot. Our workflow utilized X! Tandem to search gastropod protein sequences from GenBank and *Angiostrongylus* transcriptomic data, comparing experimental mass spectra with theoretical spectra generated from known protein sequences. A minimum of 2 unique peptides, each containing at least eight amino acids, was required to confidently confirm a protein’s identity (Nakayasu *et al.*, 2021). In parallel, ProteinPilot employed curated reference databases from UniProtKB and SwissProt, adding a further validation layer. This dual approach enhanced the robustness of protein identifications by cross-validating outputs from distinct data sources.

Quantification was performed using Sequential Window Acquisition of All Theoretical Fragment Ion Spectra (SWATH), enabling precise and reproducible measurements of protein abundance across samples (Messner et al., 2021). SWATH captures ion fragments in sequential mass windows, ensuring a comprehensive and unbiased acquisition of fragmentation data, even in complex mixtures. Stringent statistical thresholds were applied to validate our findings: only proteins with a false discovery rate of ≤2% and a *P*-value of ≤0·05 were considered significant. These rigorous filters ensure that only high-confidence, statistically robust protein identifications are reported.

## Results

A total of 103 proteins were identified in the mucus samples of infected and non-infected *Phyllocaulis* sp. slugs. In the IG, five proteins matched the *Angiostrongylus* transcriptomic data: actin, ATP synthase subunit alpha, ATP synthase subunit beta, AAA family ATPase CDC48 subfamily and the ribosomal protein S11 domain. An additional 41 proteins were matched to the *Biomphalaria glabrata* reference sequences.

Our study demonstrated that *A. cantonensis* infection induces significant proteomic alterations in the mucus of *Phyllocaulis* sp. slugs. Similar proteomic modifications have also been observed in another mollusc, *B. glabrata*, supporting the notion of conserved host-response mechanisms across molluscan species (Mendes et al., 2020). Among the proteins highlighted in our study, an F-BAR (Fes/CIP4 Homology–Bin/Amphiphysin/Rvs) domain–containing protein was particularly notable. Using NCBI (National Center for Biotechnology Information) BLASTp (*E*‐value threshold of 10^−20^), we identified homologous sequences bearing the F-BAR domain in multiple *B. glabrata* proteins, suggesting the presence of this domain in the *B. glabrata* proteome. F-BAR proteins have been implicated in active cellular responses, including membrane remodelling and reparative processes (Itoh et al., 2005; Frost et al., 2009), hinting at a possible mechanism by which these gastropod hosts adapt to *A. cantonensis* infection.

**Figure 1.**
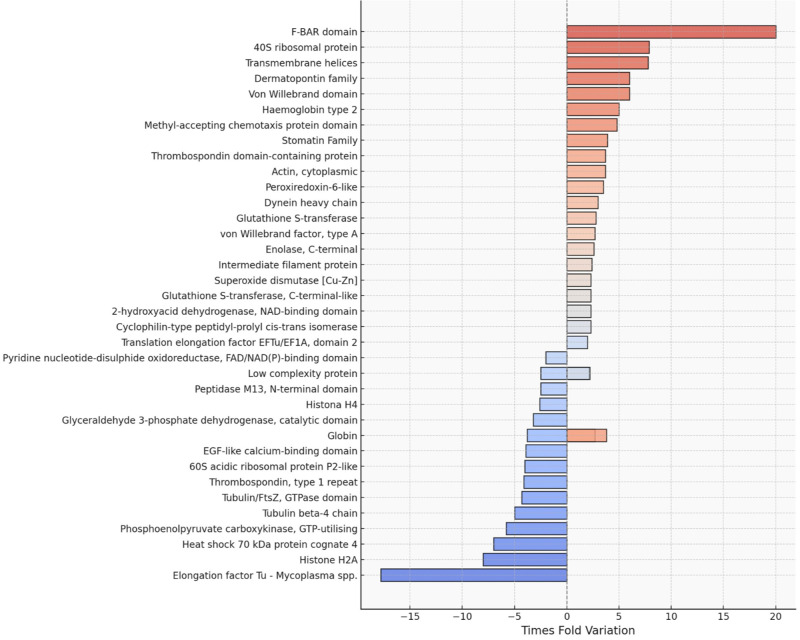
Protein expression in the mucus of *Phyllocaulis* spp. slugs infected with *Angiostrongylus cantonensis.*

Key up-regulated proteins included glycolytic enzymes such as phosphoenolpyruvate carboxykinase (PEPCK) and glyceraldehyde-3-phosphate dehydrogenase (GAPDH), as well as proteins involved in immune responses, such as von Willebrand factor type A and epidermal growth factor (EGF)-like calcium-binding domain proteins. Down-regulated proteins include histone H4, indicating potential changes in gene expression. Additionally, 2 proteins, the low-complexity protein and globin, were found to be both up- and down-regulated simultaneously (Figure 1). This observation may indicate the presence of distinct isoforms. We recommend further molecular sequence analyses of these proteins to clarify these findings.

## Discussion

Our study demonstrated that *A. cantonensis* infection induced significant proteomic alterations in the mucus of *Phyllocaulis* sp. slugs. The up-regulation of the F-BAR domain-containing protein suggests an active cellular response involving membrane remodelling and repair processes (Itoh et al., 2005; Frost et al., 2009). Similar findings in *B. glabrata* infected with *A. cantonensis* support the notion of conserved immune response mechanisms across mollusc species (Mendes et al., 2020).

The down-regulation of histone H4 indicates possible epigenetic modifications that affect gene expression in response to infection (Kornberg and Lorch, 1999; Saha et al., 2006). This aligns with previous observations in *B. glabrata*, suggesting that histone modifications may play a role in molluscan defence against parasites.

The up-regulation of glycolytic enzymes, such as PEPCK and GAPDH, reflects metabolic adjustments to meet increased energy demands during infection (Hanson and Garber, 1972; Sirover, 1999). Enhanced glycolysis may provide the necessary ATP for immune response and tissue repair.

Identifying proteins such as von Willebrand factor type A and EGF-like calcium-binding domain proteins underscores their potential as biomarkers for *A. cantonensis* infection. These proteins are involved in coagulation and cell signalling pathways, which are critical for immune functions (Ober and Litingtung, 2001; Ruggeri, 2003).

The significant down-regulation of elongation factor Tu from *Mycoplasma* sp. suggests a functional interplay between helminth infection and the mollusc microbiota. *Mycoplasma* sp. has been detected in the microbiota of both *A. cantonensis*‐infected and non-infected molluscs (Osório et al., 2020). This observation may be explained by immune-mediated processes that influence bacterial adhesion (Jonák, 2007).

Detecting *Angiostrongylus*-derived proteins in slug mucus, such as actin and ATP synthase subunits, may result from larval degradation or immune responses, leading to larval death (Bonetti and Graeff-Teixeira, 1998; Lange et al., 2017). Thus, these proteins may serve as direct indicators of infection.

The presence of lipopolysaccharide-binding proteins, such as BgLBP/BPI1 and LBP/BPI1.2, exclusively in infected mucus samples, indicates an activated innate immune response against gram-negative bacteria (Bingle and Craven, 2004; Baron et al., 2013). This may reflect a broader immune activation in response to parasitic infections.

The proteomic changes observed in the mucus of *Phyllocaulis* sp. infected with *A. cantonensis* highlight potential biomarkers for infection and provide insights into host immune and metabolic responses. The identification of up-regulated proteins involved in membrane remodelling, energy metabolism and immune function suggests active host defence mechanisms.

Future studies should focus on validating these biomarkers in larger populations and diverse mollusc species, studying possible changes among species from the same genus. Investigating the molecular pathways underlying these proteomic shifts could enhance our understanding of host–parasite interactions and support the development of non-invasive diagnostic tools for monitoring *A. cantonensis* transmission.

## References

[ref1] Baron OL, Gouvêa YB, Do Nascimento AC and Barbosa MC (2013) Parental transfer of the antimicrobial protein LBP/BPI protects *Biomphalaria glabrata* eggs against oomycete infections. *PLOS Pathogens* 9(12), e1003792. doi:10.1371/journal.ppat.1003792 24367257 PMC3868517

[ref2] Bingle CD and Craven CJ (2004) Meet the relatives: A family of BPI- and LBP-related proteins. *Trends in Immunology* 25(2), 53–55. doi:10.1016/j.it.2003.11.007 15106612

[ref3] Bonetti VCBD and Graeff-Teixeira C (1998) *Angiostrongylus costaricensis* and the intermediate hosts: Observations on elimination of L3 in the mucus and inoculation of L1 through the tegument of molluscs. *Revista Brasileira de Medicina Tropical* 31(3), 289–294. doi:10.1590/S0037-86821998000300006 9612020

[ref4] Caldeira RL, Mendonça CLGF, Goveia CO, Lenzi HL, Graeff-Teixeira C, Lima WS and Mota EM (2007) First record of molluscs naturally infected with *Angiostrongylus cantonensis* (Chen, 1935) (Nematoda: Metastrongylidae) in Brazil. *Memórias do Instituto Oswaldo Cruz* 102(7), 887–889. doi:10.1590/S0074-02762007000700018 18094889

[ref5] Chan D, Barratt J, Roberts T, Lee R, Marriott D, Harkness J and Ellis J (2015) The prevalence of *Angiostrongylus cantonensis/mackerrasae* complex in molluscs from the Sydney region. *PLoS ONE* 10(5), e0128128. doi:10.1371/journal.pone.0128128 26000568 PMC4441457

[ref6] Cognato BB, Morassutti AL, Garcia JS and Graeff-Teixeira C (2013) First report of *Angiostrongylus cantonensis* in Porto Alegre, State of Rio Grande do Sul, Southern Brazil. *Revista da Sociedade Brasileira de Medicina Tropical* 46(5), 664–665. doi:10.1590/0037-8682-0073-2013 24270262

[ref7] Frost A, Unger VM and De Camilli P (2009) The BAR domain superfamily: Membrane-molding macromolecules. *Cell* 137(2), 191–196. doi:10.1016/j.cell.2009.04.010 19379681 PMC4832598

[ref8] Graeff-Teixeira C, Thiengo SC, Lee SC and Yoshimura K (1993) On the diversity of mollusc intermediate hosts of *Angiostrongylus costaricensis* Morera & Céspedes, 1971 in southern Brazil. *Memórias do Instituto Oswaldo Cruz* 88(3), 487–489. doi:10.1590/S0074-02761993000300020 8107609

[ref9] Hanson RW and Garber AJ (1972) Phosphoenolpyruvate carboxykinase. I. Its role in gluconeogenesis. *The American Journal of Clinical Nutrition* 25(11), 1234–1251. doi:10.1093/ajcn/25.11.1234 4342753

[ref10] Itoh T, Erdmann KS, Roux A, Habermann B, Werner H and De Camilli P (2005) Dynamin and the actin cytoskeleton cooperatively regulate plasma membrane invagination by BAR and F-BAR proteins. *Developmental Cell* 9(6), 791–804. doi:10.1016/j.devcel.2005.11.005 16326391

[ref11] Jonák J (2007) Bacterial elongation factors EF-Tu, their mutants, chimeric forms, and domains: Isolation and purification. *Journal of Chromatography B* 849(1–2), 141–153. doi:10.1016/j.jchromb.2006.11.053 17197255

[ref12] Kornberg RD and Lorch Y (1999) Twenty-five years of the nucleosome, fundamental particle of the eukaryote chromosome. *Cell* 98(3), 285–294. doi:10.1016/S0092-8674(00)81958-3 10458604

[ref13] Lange MK, Penagos-Tabares F, Muñoz-Caro T, Gärtner U and Hermosilla C (2017) Gastropod-derived haemocyte extracellular traps entrap metastrongyloid larval stages of *Angiostrongylus vasorum, Aelurostrongylus Abstrusus* and *Troglostrongylus brevior*. *Parasites & Vectors* 10(1), 50. doi:10.1186/s13071-016-1961-z 28143510 PMC5282800

[ref14] Mendes LF, Araújo AM and Gomes LC (2020) Proteomic changes in *Biomphalaria glabrata* infected with *Angiostrongylus cantonensis*. *Acta Tropica* 203, 105314. doi:10.1016/j.actatropica.2020.105314 32931750

[ref15] Messner CB, Demichev V, Bloomfield N, Yu JSL, White M, Kreidl M, Egger A-S, Freiwald A, Ivosev G, Wasim F, Zelezniak A, Jürgens L, Suttorp N, Sander LE, Kurth F, Lilley KS, Mülleder M, Tate S and Ralser M (2021) Ultra-fast proteomics with Scanning SWATH. *Nature Biotechnology.* 39, 846–854. doi:10.1038/s41587-021-00860-4 PMC7611254 33767396

[ref16] Morassutti AL, Thiengo SC, Fernandez M, Sawanyawisuth K and Graeff-Teixeira C (2014) Eosinophilic meningitis caused by *Angiostrongylus cantonensis*: An emergent disease in Brazil. *Memórias do Instituto Oswaldo Cruz* 109(4), 399–407. doi:10.1590/0074-0276140023 25075779 PMC4155839

[ref17] Nakayasu ES, Gritsenko M, Piehowski PD, et al. (2021) Tutorial: best practices and considerations for mass-spectrometry-based protein biomarker discovery and validation. *Nature Protocols* 16(8), 3737–3760. doi:10.1038/s41596-021-00566-6 34244696 PMC8830262

[ref18] Ober EA and Litingtung Y (2001) The role of the EGF-CFC genes in the development of vertebrate organs. *Development* 128(18), 3863–3874. doi:10.1242/dev.128.18.3863

[ref19] Osório JB, de Mattos Pereira L, Giongo A, et al. (2020) Mollusk microbiota shift during *Angiostrongylus cantonensis* infection in the freshwater snail *Biomphalaria glabrata* and the terrestrial slug *Phillocaulis soleiformis*. *Parasitology Research* 119, 2495–2503. doi:10.1007/s00436-020-06743-y 32556501

[ref20] Potriquet J, Laohaviroj M, Bethony JM and Mulvenna J (2017) A modified FASP protocol for high-throughput preparation of protein samples for mass spectrometry. *PLoS ONE* 12(7), e0175967. doi:10.1371/journal.pone.0175967 28750034 PMC5531558

[ref21] Qvarnstrom Y, Xayavong M, da Silva ACA, Park SY, Whelen AC, Calimlim PS and da Silva AJ (2007) PCR-based detection of *Angiostrongylus cantonensis* in tissue and mucus secretions from molluscan hosts. *Applied and Environmental Microbiology* 73(5), 1415–1419. doi:10.1128/AEM.01968-06 17194836 PMC1828786

[ref22] Rizzi V, Gubitosa J, Fini P and Nuzzo S (2021) Snail slime-based gold nanoparticles: An interesting potential ingredient in cosmetics as an antioxidant, sunscreen, and tyrosinase inhibitor. *Journal of Photochemistry & Photobiology, B: Biology* 224, 112309. doi:10.1016/j.jphotobiol.2021.112309 34563935

[ref23] Ruggeri ZM (2003) Von Willebrand factor, platelets and endothelial cell interactions. *Journal of Thrombosis and Haemostasis* 1(7), 1335–1342. doi:10.1046/j.1538-7836.2003.00260.x 12871266

[ref24] Saha A, Wittmeyer J and Cairns BR (2006) Chromatin remodelling: The industrial revolution of DNA around histones. *Nature Reviews Molecular Cell Biology* 7(6), 437–447. doi:10.1038/nrm1945 16723979

[ref25] Sirover MA (1999) New insights into an old protein: The functional diversity of mammalian glyceraldehyde-3-phosphate dehydrogenase. *Biochimica et Biophysica Acta (BBA) - Protein Structure and Molecular Enzymology* 1432(2), 159–184. doi:10.1590/S0074-02761993000300020 10407139

[ref26] Tsvetanova E, Alexandrova A, Georgieva A and Miteva K (2020) Effect of mucus extract of *Helix aspersa* on scopolamine-induced cognitive impairment and oxidative stress in rat’s brain. *Bulgarian Chemical Communications* 52, 107–111.

[ref27] Wang Q-P, Wu Z-D, Wei J, Owen RL and Lun Z-R (2012) Human *Angiostrongylus cantonensis*: An update. *European Journal of Clinical Microbiology & Infectious Diseases* 31(4), 389–395. doi:10.1007/s10096-011-1328-5 21725905

